# GIP Contributions to the Regulation of Centromere at the Interface Between the Nuclear Envelope and the Nucleoplasm

**DOI:** 10.3389/fpls.2016.00118

**Published:** 2016-02-08

**Authors:** Marie-Edith Chabouté, Alexandre Berr

**Affiliations:** Institut de biologie moléculaire des plantes, CNRS, Université de StrasbourgStrasbourg, France

**Keywords:** centromere assembly, centromere maintenance, replication, cohesion, *Arabidopsis*, GIP, nuclear envelope

## Abstract

Centromeres are known as specific chromatin domains without which eukaryotic cells cannot divide properly during mitosis. Despite the considerable efforts to understand the centromere/kinetochore assembly during mitosis, until recently, comparatively few studies have dealt with the regulation of centromere during interphase. Here, we briefly review and discuss past and recent advances about the architecture of centromeres and their regulation during the cell cycle. Furthermore, we highlight and discuss new findings and hypotheses regarding the specific regulation of centromeres in both plant and animal nuclei, especially with GIP proteins at the interface between the nuclear envelope and the nucleoplasm.

## Introduction

Originally, the regions where spindle fibers attach to each chromosome during cell division were named “centromere” (Darlington, 1936) and “kinetochore” (Schrader, 1936) and were long thought redundant. Nowadays, functional centromeres are generally recognized as specialized chromosome regions involved in promoting sister chromatid cohesion, where DNA interacts with kinetochore components to ensure the faithful segregation of genetic material during mitosis and meiosis (Steiner and Henikoff, 2014). Centromeres are shaped and regulated by genetic, molecular, and epigenetic mechanisms. With a few exceptions (e.g., the point centromeres of *Saccharomyces cerevisiae* and the centromeres of at least one species of the parasitic protozoan *Trypanosoma*; Clarke and Carbon, 1980; Obado et al., 2007), nearly all centromeres comprise highly homogeneous repetitive arrays of DNA satellites. Indeed, in multicellular eukaryotic species, centromeres are enriched in long stretches of 1000s of copies of species-specific repetitive satellites, ranging in length from ∼150 to ∼210 bases (i.e., in the order of magnitude required to form a single nucleosome). The discordance between the highly conserved function of centromeres among eukaryotes and the absence of a universal DNA sequence is one of the greatest conundrums in eukaryotic biology and is better known as the “centromere paradox” (Henikoff et al., 2001). Based on sequences and protein content, a centromere can be separated into two domains, (i) the inner/core domain containing highly homogenized satellite arrays and no/few insertions of transposable elements (i.e., namely transposons and/or retrotransposons) and (ii) the outer/flanking domain which interacts with microtubules to form spindles and which contains diverging satellite arrays and significantly more inserted elements (Plohl et al., 2014).

Basically, the life cycle of a somatic cell can be subdivided into two alternating phases, a longer one named interphase (i.e., further subdivided in G1, S, and G2 phases) and a much shorter one named mitosis. Kinetochore/centromere organization, function and regulation, including microtubule binding, chromosome movement, and also checkpoint signaling, are beginning to be well understood, especially during mitosis and have been reviewed recently (see Fukagawa and Earnshaw, 2014; Auckland and McAinsh, 2015; Lermontova et al., 2015; Marston, 2015). In addition, it appears that nuclear envelope components, such as nuclear pore proteins, are critical at kinetochores for accurate chromosome segregation during mitosis in both animals and plants (for reviews, see Boruc et al., 2012; Ibarra and Hetzer, 2015). Conversely, our understanding of the regulation of centromeres especially during interphase remains more elusive. While connection of centromere with nuclear envelope and microtubules is well established in *S. pombe* to control cell division (King et al., 2008; Hou et al., 2012), it still remains poorly understood in plants even though centromeres are embedded in the pericentromeric heterochromatin at the nuclear periphery (Fransz et al., 2002). Looking for regulators of microtubule nucleation at the nuclear envelope, we discovered the small 8 kDa GCP3-interacting proteins GIP1 and GIP2 in *Arabidopsis* (Janski et al., 2008, 2012). These alpha helix proteins can homo-heterodimerize (Batzenschlager et al., 2013), and may be involved in the regulation of different cellular processes according to the pleiotropic developmental phenotypes of the *gip1 gip2* double mutant (Janski et al., 2012). Furthermore, GIPs are located on both sides of the nuclear envelope, they favor the anchoring of the nucleation complexes at the outer nuclear membrane, and are closely associated with centromeres at the inner nuclear membrane (Batzenschlager et al., 2013).

In this review, we discuss recent progress in understanding the organization and regulation of centromeres during this phase with a special emphasis on newly identified proteins, such as GIPs at the interface between the nuclear envelope and the nucleoplasm.

### Update on Centromere Assembly and Maintenance: New Key Factors Such as GIPs Proteins

Beside its highly repetitive DNA sequence, another feature of the centromeric core/inner domain (also considered as the most prominent protein identifier of the centromere function) is the centromere- and species-specific histone variant called CENP-A in mammals (Earnshaw and Rothfield, 1985), Cse4 in *Saccharomyces cerevisiae* (Meluh et al., 1998), Cnp1 in *S. pombe* (Takahashi et al., 2000), CID in fruit flies (Henikoff et al., 2000) and CENH3 in plants (Jiang et al., 2003), which replaces histone H3 in a proportion of nucleosomes in centromeric chromatin. Following a unified nomenclature across species, we will simply refer to this protein as CENH3 (Talbert and Henikoff, 2013). Despite the occasional occurrence of neo/*de novo* centromeres in both plant and animal species (Dawe and Hiatt, 2004; Marshall et al., 2008), there may be a certain dependency of the centromere function upon the DNA sequence and/or organization of the centromeric region, as centromeres are usually maintained at the same loci from generation to generation. In mammals and *Drosophila*, CENH3 deposition is uncoupled from centromeric DNA replication as it occurs after (i.e., during G1) or during mitosis, respectively (Hemmerich et al., 2008; Mellone et al., 2011). Using mouse cultured cell lines, the amount of CENH3 deposition at centromeres was recently found to be controlled by the pericentromeric heterochromatin integrity and linked to structural rearrangement (i.e., replacement of H2A by the H2A.Z variant) of the pericentromeric region in the G1 phase (Boyarchuk et al., 2014). This finding perfectly illustrates the importance of the crosstalk between centric and pericentric domains for centromere assembly throughout the cell cycle. In fission yeast and plants, newly synthesized CENH3 is loaded at centromeres during the S/G2 phases or late G2 phase, respectively, suggesting that CENH3 incorporation and kinetochore formation occur at the same time (Lermontova et al., 2006; Dunleavy et al., 2007). Thus, it appears that kinetochore assembly and chromosome segregation occur with only half of the maximal amount of CENH3 present on one of the separated sister chromatids in telophase/G1 in mammals and *Drosophila*, while plants and fungi possess the full amount of CENH3 already at G2/prophase leading to the assumption that the pre-divisional CENH3 recruitment was probably acquired as the ancestral mechanism during evolution of eukaryotes (Dubin et al., 2010; Schubert et al., 2014).

In the last decade, much progress has also been made concerning the CENH3 deposition and maintenance machinery, with the identification of specific complexes: (i) the MIS18 complex (i.e., composed, among other proteins, of MIS18BP/KNL2; reviewed in Stellfox et al., 2013) was found to be necessary to initiate centromere assembly, and (ii) CENH3 chaperones, such as HJURP in mammals, Scm3 in fission yeast, Sim3 in budding yeast (reviewed in De Rop et al., 2012) or more recently CAL1 in *Drosophila* (Chen et al., 2014), were found to be required for CENH3 deposition at centromeres. Each of these chaperones was shown to selectively bind CENH3, and not the canonical H3, and to mediate the formation of CENH3 nucleosomes *in vitro*. Similarly, in *Arabidopsis*, the KNL2 homolog was proposed to be involved in CENH3 loading at centromeres (Lermontova et al., 2013).

More recently, new knowledge on centromere assembly/maintenance emerged with the characterization of GIP originally identified in eukaryotes using the plant model *Arabidopsis thaliana* (Janski et al., 2008). As being highly conserved among eukaryotes (Batzenschlager et al., 2013), GIP protein homologs were later characterized in humans and *S. pombe* (for a review, see Teixidó-Travesa et al., 2012). Besides their roles in the establishment of a robust spindle during mitosis in *Arabidopsis* (Janski et al., 2012), GIPs also play a key role in the CENH3 loading and maintenance (Batzenschlager et al., 2015). Interestingly, while less CENH3 is recruited at the centromere in both the *gip1gip2* and the *knl2* mutants, the KNL2 protein is mislocated and its level increased in *gip1gip2* mutants’ chromatin.

In parallel to the identification of potential direct/indirect CENH3 chaperones, several studies in multiple organisms have pointed out the importance of centromere transcription for the maintenance of centromere integrity (reviewed in Gent and Dawe, 2012; Hall et al., 2012; Scott, 2013). In humans, centromeric transcription is required for HJURP and CENH3 targeting at centromeres (Quenet and Dalal, 2014). Recently, it was also proposed that both sequence features and transcriptional stalling within centromere DNA promote establishment of CENH3 chromatin in *S. pombe* (Catania et al., 2015). Similarly, in maize and *Arabidopsis*, centromeric transcripts were involved as structural and regulatory components of centromeres (Topp et al., 2004; May et al., 2005). Although many gaps in our knowledge remain to be filled, centromeric RNAs seem to act as scaffolds for the recruitment and the organization of key centromeric proteins such as CENH3.

Together, these works suggest an evolutionarily conserved basis for the phenomenon of CENH3 loading at centromeres, in both animals and plants. However, the interplay between GIP and KNL2 proteins for CENH3 loading remains to be established.

### Chromosome Architecture During Interphase: GIPs as a Link Between the Nuclear Envelope and the Centromeric Scaffold

Inside the cell interphase nucleus, the eukaryotic genome is territorially organized in discrete chromosome territories (CTs), which generally do not overlap. Genes inside their respective CTs may be regulated by chromatin looping and long-range inter-chromosomal interactions (reviewed in Deng and Blobel, 2014; Guo and Fang, 2014). Such an organization of the genome is recognized to play fundamental regulatory roles in all cellular functions that involve DNA clustering of chromosome regions within interphase nuclei is supposed to be an important mechanism regulating the functional architecture of chromatin. Indeed, gene expression is correlated with the relative position of genes to different nuclear domains, such as the nuclear envelope, the nucleolus, or some heterochromatic clusters (reviewed in Gartenberg et al., 2004; Fraser and Bickmore, 2007; Brickner, 2009; Towbin et al., 2012).

Inside CTs, centromeres occupy specific positions and can aggregate in distinct chromocenters. In mammalian cells, the positions of centromeres (and pericentromeric heterochromatin) are non-random and characteristic of each cell type. In human cells, depending on the cell cycle phase, centromeres can either be located at the nuclear periphery or more internally around the nucleolus (Solovei et al., 2004). So far, the mechanisms involved in this dynamic pattern and its functional impact have not yet been elucidated. An alternative to this radial organization is the polarized Rabl configuration in which centromeres and telomeres are positioned facing opposite poles of the nucleus (for review, see Schubert and Shaw, 2011). A Rabl-like organization, described for *Trypanosoma*, yeast, salamander, and *Drosophila*, is also commonly found in plants (e.g., in wheat, rye, barley, and onion root tip cells). The significance of such an organization is still much debated and this configuration might finally reflect the lack of reorganization of centromere and telomere after telophase when the nuclear envelope reforms. In *Arabidopsis*, the Rabl configuration does not last after mitosis. Indeed, in interphasic nuclei centromeres/chromocenters are located at the nuclear periphery, while telomeres are often found around the nucleolus (Fransz and de Jong, 2011).

Inside the nuclear envelope in animals, plants and yeast, specialized Linker of Nucleoskeleton and Cytoskeleton (LINC) complexes, composed among others of Sad1/UNC-84 (SUN)-domain proteins, physically connecting the cytoskeleton to the nucleoskeleton were involved in multiple functions (reviewed in Chang et al., 2015; Kim et al., 2015; Zhou et al., 2015). In fission yeast, the inner nuclear membrane SUN domain protein Sad1 and the nucleoplasmic adaptor Csi1 connect centromeres to the nuclear envelope during interphase (Guo and Fang, 2014). Interestingly, this clustering was found to be critical for the efficient capture of kinetochores by microtubules in early mitosis (Hou et al., 2012). In budding yeast, the SUN protein Mps3 is required for the insertion into the nuclear membrane of the spindle pole body (i.e., the microtubule organizing center in yeast cells, functionally equivalent to the centrosome; Friederichs et al., 2011). Short kinetochore microtubules were found to link each centromere to the spindle pole body in a kind of “rosette” (Laporte et al., 2013). While, such clustering seems involved in the nuclear morphology and the chromatin organization (Ramdas and Shivashankar, 2015), whether it is important for the fidelity of chromosome segregation in the subsequent anaphase is still an open question.

Linker of Nucleoskeleton and Cytoskeleton complexes are conserved in eukaryotes (Zhou and Meier, 2013), however, there was until now no evidence that a link between the nuclear envelope and the centromeric scaffold may exist in plants. Besides the role of GIPs in stabilizing the microtubule nucleation complexes at the nucleation sites (Janski et al., 2012), we recently demonstrated that GIPs co-immunoprecipitate with CENH3 and colocalize with chromocenters and CENH3 at the nuclear periphery in *Arabidopsis* interphase nuclei (Batzenschlager et al., 2015). The CROWDED NUCLEI (CRWN/NMCP/LINC) proteins were suggested to work as functional equivalent of lamins in plants (Ciska and Moreno Díaz de la Espina, 2013, 2014). Indeed, both CRWN1 and CRWN4 localize at the nuclear periphery and regulate the nuclear morphology (Sakamoto and Takagi, 2013). While CRWN1 was shown to interact with the inner nuclear membrane proteins SUN1/2 in *Arabidopsis* (Graumann, 2014), CRWN4 was involved in the maintenance of the interphase chromocenter integrity and organization (Wang et al., 2013). These facts, put in perspective with the roles played by GIPs in both nuclear shaping and nuclear envelope organization (Batzenschlager et al., 2013, 2014), suggest that a GIPs dependent-mechanism, probably involving some nuclear matrix components and in connection with the microtubule network, might be involved in the regulation of centromeres positioning and activity at the nuclear periphery. Also, it is worth to note that in mammalian cells there is evidence for the involvement of microtubules and LINC complexes in the greater mobility of chromatin surrounding double-strand breaks and DNA repair (Lottersberger et al., 2015). Such evidence is so far missing in plants.

### GIP in Centromere Cohesion, a New Field of Investigation Emerging from Plant

Cohesins mediate cohesion between sister chromatids as a prerequisite for correct sister chromatid segregation during cell division. Functional centromeres are associated with pericentromeric regions which support a strong sister chromatid cohesion to prevent premature chromatid separation (Allshire and Karpen, 2008). Except for budding yeast, which lacks pericentromeric heterochromatin, in most organisms, pericentromeric heterochromatin is characterized by di- and trimethylation of histone H3 on lysine 9 (reviewed in Birchler and Han, 2013; Padeganeh et al., 2013). In most eukaryotes, the heterochromatin protein 1 (HP1) binds to pericentromeric heterochromatin, thus contributing to the recruitment of shugoshin involved in the protection of the centromere. Mounting evidence in fission yeast and mammals suggests that the localization of shugoshin during interphase might be required for maintaining pericentromeric and centromeric cohesion until the onset of anaphase (for review, see Marston, 2015). Interestingly, this protection of centromere by shugoshin is not conserved in *Arabidopsis* somatic cells (Zamariola et al., 2014). Moreover, LHP1/TFL2, the unique *Arabidopsis* HP1 homolog known so far, is exclusively located in euchromatin (Turck et al., 2007) and does not co-localize with the heterochromatic chromocenter (Nakahigashi et al., 2005). Our recent work has identified GIPs at the nuclear periphery and highlights the significant contribution of GIPs to maintain centromeric cohesion. Forty percent of root tip 4C nuclei in our *gip1 gip2* double mutant showed more than 10 centromeric signals and an increased intercentromere distance (Batzenschlager et al., 2015). This result suggests that GIPs in plants may function as shugoshin equivalents. Interestingly, the plant specific protein PATRONUS1 (PANS1) was also suggested to contribute to centromere cohesion maintenance in somatic cells, to a lower extent than GIPs (i.e., only 10% of root tip nuclei in the *pans1* mutant showed more than 10 centromeric signals; Zamariola et al., 2014). Together, these data pinpoint GIP and PANS1 as important regulators of centromere cohesion in *Arabidopsis*, whose underlying mechanisms need to be deciphered.

### GIPs Proteins, a Cornerstone at the Nucleo-Cytoplasmic Interface for Multiple Nuclear Functions?

In yeast and human, CTF7/Eco1 is essential during S phase to establish cohesion throughout acetylation of SMC3 and also to regulate replication fork progression (for review, see Rudra and Skibbens, 2013). Moreover, in fission yeast, early replication of pericentromeric heterochromatin is linked to the robust pericentromeric cohesion (Tanaka et al., 2013) and may be coordinated by kinetochores (Natsume et al., 2013). Together, these findings provide new insights into the relationship between DNA replication and cohesion establishment. Since both *CTF7* transcript level and centromere/pericentromere cohesion were found decreased in the *gip1gip2* mutant showing aneuploidy (Batzenschlager et al., 2015), it still remains to be established how GIPs can function at the interface between cohesion and replication from G1/S to S phases.

**FIGURE 1:**
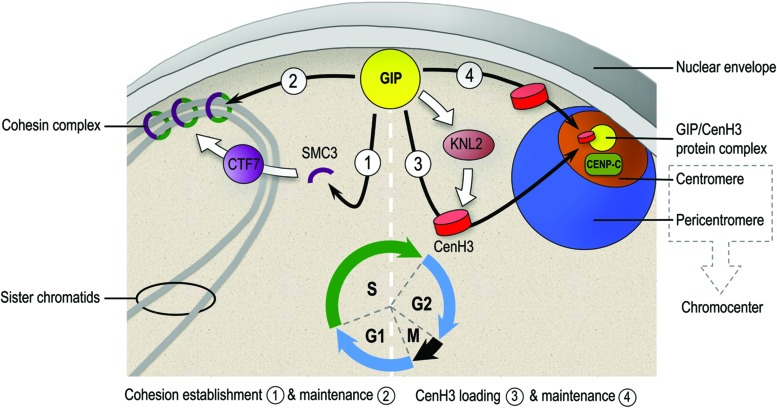
**Hypothetical model depicting the different roles played by GIPs at the nuclear envelope and nucleoplasm interface for centromere regulation in *Arabidopsis thaliana*.** During the G1/S phase, GIPs may contribute to the cohesion establishment/maintenance at centromeres probably through a CTF7/SMC3-mediated mechanism. During the G2 phase, GIPs may participate to the loading/maintenance of CENH3 at centromeres probably through a regulatory pathway complementary to the one proposed for KNL2 (Lermontova et al., 2013). Together, GIPs seem to operate as a multifunctional hub at the nuclear envelope periphery to coordinate centromeric functions and establish with CENP-C a functional kinetochore essential for chromosome segregation.

Very recently, CENP-C was proposed as a key factor connecting kinetochore assembly to CENP-A loading (Shono et al., 2015). Because CENP-C is decreased in the *gip* double mutant, it is tempting to speculate that in plants GIP may facilitate the deposition and/or maintenance of CENH3 in G2 for proper kinetochore assembly at the nuclear envelope (**Figure 1**). Due to the conservation of GIP, CENH3, and KNL2 proteins in eukaryotes, we speculate that in both plants and animals, the accurate CENH3 targeting at centromeres probably requires a “dual-lock” system: (i) chromatin-bound centromeric factors, such as the Mis18 complex including KNL2 establish the centromeric epigenetic boundary for the recruitment of CENH3 and (ii) a CENH3-chaperone complex regulated by and/or including GIPs effectively targets CENH3 to pre-existing active centromeric sites.

The functional characterization of GIPs at the nucleo-cytoplasmic interface has highlighted their multiple roles in the regulation of centromeres along the cell cycle in *Arabidopsis* (Janski et al., 2012; Batzenschlager et al., 2013, 2015). Because GIPs are conserved among eukaryotes and are also named MOZART1/MZT1 (mitotic-spindle organizing protein 1), our findings may be relevant to future studies in other organisms. Also, related to the recent findings that modulating chromatin flow can define both transient and long-lived changes in nuclear shape (Schreiner et al., 2015), our data at the nucleo-cytoplasmic interface may open new fields of investigation of GIPs functions in response to cytoskeletal forces and nuclear mechanics.

## Author Contributions

AB and M-EC wrote the mini-review. AB created the figure.

## Conflict of Interest Statement

The authors declare that the research was conducted in the absence of any commercial or financial relationships that could be construed as a potential conflict of interest.
